# Long-Term Mechanical Behavior of Nano Silica Sol Grouting

**DOI:** 10.3390/nano8010046

**Published:** 2018-01-16

**Authors:** Dongjiang Pan, Nong Zhang, Chenghao Zhang, Deyu Qian, Changliang Han, Sen Yang

**Affiliations:** 1 Key Laboratory of Deep Coal Resource Mining, School of Mines, China University of Mining and Technology, Xuzhou 221116, China; cumtpdj@163.com (D.P.); zhangchenghao421@126.com (C.Z.); hclayor@126.com (C.H.); yangsen91811@163.com (S.Y.); 2 Department of Energy and Mineral Engineering, G3 Center and Energy Institute, Pennsylvania State University, PA 16802, USA

**Keywords:** nano silica sol, long-term mechanical tests, fluctuating temperature-humidity conditions, micro-mechanism

## Abstract

The longevity of grouting has a significant effect on the safe and sustainable operation of many engineering projects. A 500-day experiment was carried out to study the long-term mechanical behavior of nano silica sol grouting. The nano silica sol was activated with different proportions of a NaCl catalyst and cured under fluctuating temperature and humidity conditions. The mechanical parameters of the grout samples were tested using an electrohydraulic uniaxial compression tester and an improved Vicat instrument. Scanning electron microscope, X-ray diffraction, and ultrasonic velocity tests were carried out to analyze the strength change micro-mechanism. Tests showed that as the catalyst dosage in the grout mix is decreased, the curves on the graphs showing changes in the weight and geometric parameters of the samples over time could be divided into three stages, a shrinkage stage, a stable stage, and a second shrinkage stage. The catalyst improved the stability of the samples and reduced moisture loss. Temperature rise was also a driving force for moisture loss. Uniaxial compressive stress-strain curves for all of the samples were elastoplastic. The curves for uniaxial compression strength and secant modulus plotted against time could be divided into three stages. Sample brittleness increased with time and the brittleness index increased with higher catalyst dosages in the latter part of the curing time. Plastic strength-time curves exhibit allometric scaling. Curing conditions mainly affect the compactness, and then affect the strength.

## 1. Introduction

Nano silica sol is a kind of grouting material made up of nanometer particles that is highly transmissive [1]; it has been used in the geotechnical field in recent years [2]. Jurinak [3] developed a practical fluid-flow control system based on colloidal silica gel for oilfield reservoirs. Persoff et al. [4] studied permanent barrier systems formed by colloidal silica and polysiloxane for contaminant isolation. Saito et al. [5] proposed the use of silica sol to maintain overburden stability during shield tunneling. Butron et al. [6,7] tested the mechanical properties and failure of silica sol used for rock grouting for periods of up to half a year. Funehag et al. [8,9,10] designed a grouting procedure for a tunnel using silica sol. McCartney et al. [11] evaluated the formation of hydraulic barriers for secondary containment through the permeation of colloidal silica grout. Gallagher et al. [12] studied a passive site stabilization technique for nondisruptive mitigation of liquefaction risk using colloidal silica. Wang et al. [13] used silica sol to seal phreatic pore water and fractured confined groundwater in the Xiao Jihan Coal Mine, China.

Because nano silica sol is hydrolyzed mineral slurry, both eco-friendly and non-polluting, it has a promising future for use in underground reservoir storage, CO_2_ storage, nuclear waste storage, and for use as a sealant in dams, mines, and other engineered structures.

Although the service lives of geotechnical engineering structures can be very long, abundant data on the long-term mechanical properties of nano silica sol are still lacking. However, the longevity of grouting has a significant effect on the safe and sustainable operation of many engineering projects. For silica sol, the influence of the environment in which the sol cures, and the proportion of catalyst used on the long-term mechanical properties of the sol are important. Butrón et al. [6] studied the strength of different batches of silica sol after curing it under different temperatures and relative humidities for 6 months. However, these experiments were not long enough for many engineering purposes because the durability of grouting projects is commonly evaluated for three time periods. These are short-term, one–two months, medium term, two months to one year, and long term, and one year to three years or longer [14].

For this paper, the environmental conditions used to test nano silica sol curing simulated those present in an underground coal mine. The periodic variations of temperature and humidity in the atmosphere on the surface directly affect the microclimate in a mine [15,16]. Influenced by the temperature fluctuations of the mine airflow, the rock surrounding an underground roadway also experiences periodic temperature variations. This temperature field can have a radius of up to 30 or 40 m. Owing to high humidity in the return air, evaporation of water, and other causes, a relatively large number of roadways can experience high humidity for months at a time. To study how the mechanical properties of nano silica sol mixed with different proportions of catalyst change over the long term in an environment of fluctuating temperature and humidity, a 500-day test of the nano silica sol’s mechanical properties was performed. Simultaneously, Scanning electron microscope (SEM), X-ray diffraction (XRD), and ultrasonic velocity tests were carried out to analyze the strength change micro-mechanism of samples. The objectives of this work are to evaluate the long-term stability of silica gel and the further measures can be taken to prolong the stability based on the study.

## 2. Materials and Methods

### 2.1. Materials

**Figure 1:**
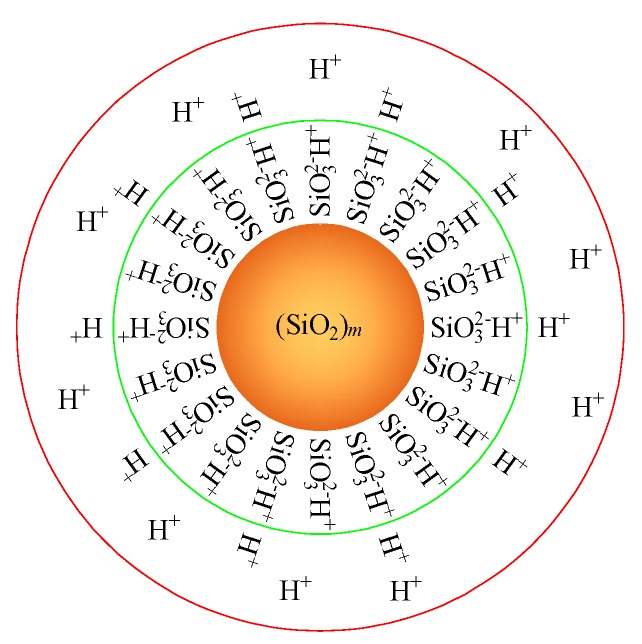
Electric double layer structure of nano silica sol.

Silica sol is a stable liquid containing individual silica particles 8–12 nm in diameter [17]. The silica sol used for this study was provided by BASF HOCK Mining Chemical (China) Company Limited (Jining, China). Silica sol has an electric double layer structure, as shown in Figure 1. When ionic sodium is added, the electric double layer becomes thinner and the silica particles connect to form a gel. Some of the physical and chemical parameters [2,17] of the silica sol and NaCl catalyst are listed in Table 1. Gel time of silica sol to catalyst of 4:1, 7:1, and 9:1 (by volume) is 10 min, 2.67 h, and 9.33 h, respectively.

### 2.2. Sample Preparation and Curing Conditions

The samples to be cured are prepared with volumetric proportions of silica sol to catalyst of 4:1, 7:1, and 9:1. The silica sol and catalyst are blended at a mixing speed of 100 revolutions per minute for 90 s utilizing RW 20 digital overhead stirrer (IKA^®^ Works Guangzhou, Guangzhou, China). The mixture is poured into a mold, a 100 mm long transparent acrylic tube with an inner diameter of 50 mm. The next day, the samples are unmolded and are put into purpose-made curing boxes.

**Figure 2:**
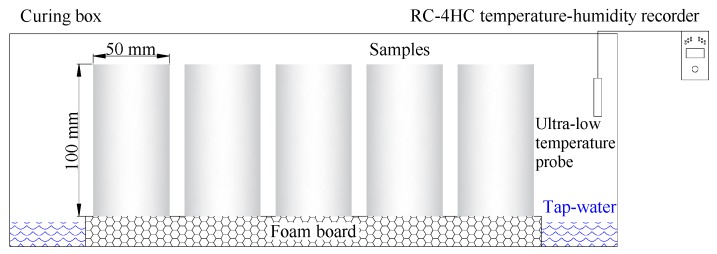
Schematic diagram of the samples, temperature-humidity recorder, and curing box used for this study. Water is added every month to maintain the depth of the water layer at 5 mm.

The curing boxes are designed to simulate the temperature and humidity of the rock surrounding a roadway, mimicking the high humidity and periodic temperature fluctuations in an underground mine. Water is poured into the bottom of each curing box and is replenished regularly to ensure a water depth of 5 mm. A foam board is immersed in the water and the samples are placed on the foam board. The temperature of the curing boxes is in natural state of a lab. An RC-4HC temperature-humidity recorder (Hangzhou SinoMeasure Automation technology Co., Ltd., Hangzhou, China) is used to monitor and record the temperature and relative humidity in the curing box. A schematic diagram of the samples, the temperature-humidity recorder, and a curing box is shown in Figure 2.

**Figure 3:**
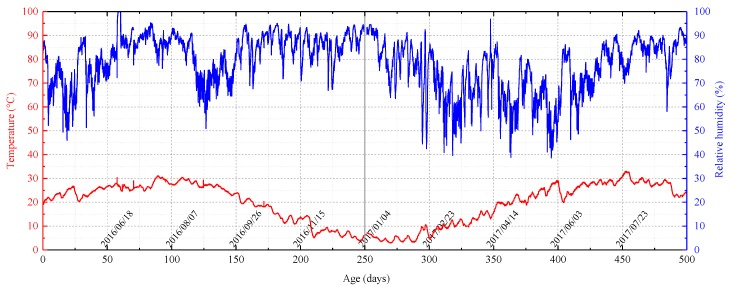
Temperature and relative humidity in the curing boxes between 29 April 2016 and 11 September 2017 (500 days). The temperature and humidity data show both long- and short-period fluctuations. The relative humidity was maintained at 50–95% and for much of the time was between 70% and 90%.

The samples were prepared on 29 April 2016. Temperature and relative humidity data from the preparation date to 11 September 2017 (500 days) are shown in Figure 3.

The temperature and humidity fluctuated over both long and short periods. For the short period fluctuations, the temperature changes are opposite those in the relative humidity. Overall, the relative humidity was maintained at 50–95% and humidity was relatively high, 70–90%, most of the time. The experiment was performed in Xuzhou City, lat 34.26° N long 117.20° E. The average daily temperature there is lower between November and February and higher between June and September. For the time intervals 0–150 days and 400–500 days of the test, the average daily temperature is usually higher than 20 °C; for the interval 200–400 days, the temperature is usually lower than 20 °C.

### 2.3. Testing Methods

The samples are weighed regularly on a precise electronic balance, the diameter and height measured with a vernier caliper, and the sample volumes calculated. The uniaxial compression strength (UCS), peak strain, secant modulus E_50_, pre-peak absorption strain energy density (PASED), and other indexes are obtained regularly by uniaxial compression tests using a UTM5504 electrohydraulic servo test machine (Shenzhen Suns Technology Stock Co., Ltd., Shenzhen, China).

UCS and peak strain reflect the ability to resist damage; PASED is the internal force that causes sudden failure of a material. *E*_50_ is the ratio of the stress at one-half of the UCS to the corresponding strain. The ratio of UCS to peak strain is denoted as *E*_p_. *E*_50_, and *E*_p_ both reflect the ability of a material to resist deformation. Nonlinear elasticity is expressed by the elastic modulus gradient, (*E*_50_ − *E*_p_)/*E*_50_). The ratio of strain energy density before and after peak stress defines the brittleness of the material. This ratio is called the brittleness index.

The ultimate shear stress (also known as plastic strength) of the grouting concretion was measured with an improved Vicat instrument (Beijing, China). Plastic strength, *P*_s_, can be determined according to the equilibrium force relationships shown in Figure 3. The value of *P*_s_ can be calculated from Equation (1) [7,18]:

(1) $$Ps=\frac{G\cos^{2}\frac{\alpha }{2}\cot\frac{\alpha }{2}}{\pi \times h^{2}}$$

where *P*_s_ is the plastic strength of the grouting concretion during the solidification of the slurry, *G* is the total weight of the Vicat cone, *α* is the conical vertex angle, and *h* is the cone penetration depth.

## 3. Results

**Figure 4:**
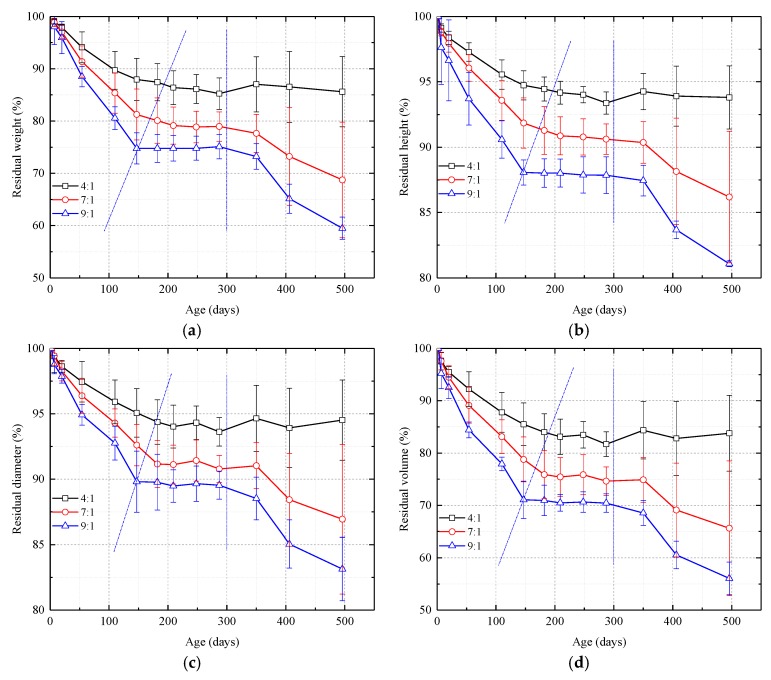
Graphs showing weight and geometric parameters vs. time for samples with different silica sol:catalyst ratios. (**a**) Residual weight; (**b**) Residual height; (**c**) Residual diameter; (**d**) Residual volume. The curves can be divided into three stages and the graphs show that the lower the amount of catalyst, the more pronounced the stages.

As shown in Figure 4, overall, the weight- and geometric parameter-time curves can be divided into three stages, a shrinkage stage, a stable stage, and a second shrinkage stage. Note that the three stages are more pronounced the lower the amount of catalyst used in the grout (or the higher the sol: catalyst ratio). In the first shrinkage stage, the weight, height, diameter, and volume of the sample decrease approximately linearly. The catalyst can apparently decrease the shrinkage rate and prolong the shrinkage stage. The graphs in Figure 4 also show that the catalyst enhances the stability of the samples and reduces moisture loss. In the stable stage, the weight and the geometric parameters remain almost unchanged. Because the stable stage takes place in the winter (Figure 2), the temperature is mainly around 8 °C and the lower temperature inhibits the sublimation of moisture. The smaller the amount of catalyst, the lower the stability. During the second shrinkage stage, the temperature has increased to 20–25 °C, enhancing moisture loss. During this stage, the weight and height of 4:1 samples decrease slightly, although the volume and diameter increases slightly. In general, the weight and geometric parameters for the 4:1 samples remain almost unchanged. The 7:1 and 9:1 samples, however, begin to shrink again although the shrinkage rate is slightly slower than during the first shrinkage stage.

**Figure 5:**
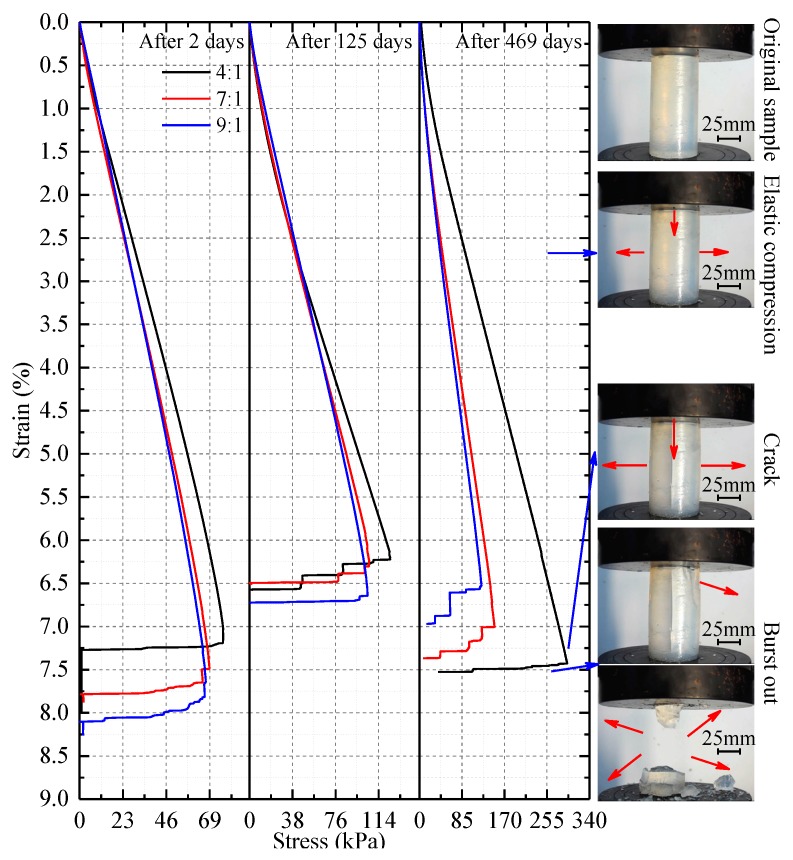
Uniaxial compressive stress-strain curves for samples with different proportions of silica sol and catalyst at three different times during the course of the experiment. The curves show that all the samples behaved as elastoplastic solids.

Figure 5 shows that the uniaxial compressive stress-strain curves for samples with all three silica sol:catalyst ratios are elastoplastic no matter the sample’s age. The deformation can be divided into four phases. These are (1) a compaction phase at strains of 0% to 1%. The deduction is that the weight and volume decrease causing internal micropores to be generated and the micropores are then closed by the external forces. (2) An elastic phase during which the stress strain curve is approximately linear. This phase endures over a great range of strain percentages. (3) A plastic phase during which a crack is initiated in the sample and rapidly develops. The crack propagates and the sample is damaged by I-shaped tensile fracture. This stage is relatively short. (4) The post-peak stress phase, the sample is suddenly broken into pieces and flies apart almost instantaneously.

**Figure 6:**
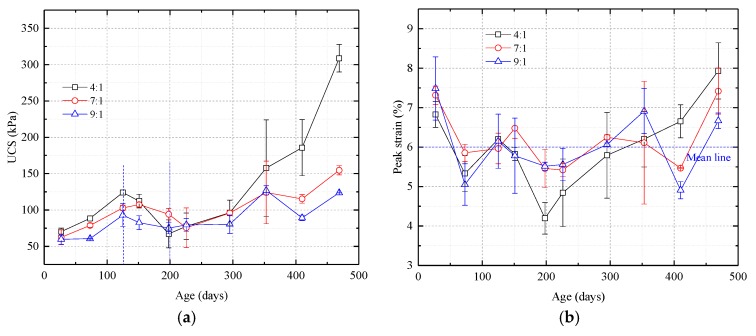
Uniaxial compression strength and peak strain vs. time curves for samples with different proportions of silica sol and catalyst. (**a**) UCS curves. The curves can be divided into an ascending stage, a descending stage, and a second ascending stage; (**b**) Peak strain curves. Note that the sample’s age and the proportion of catalyst have little effect on the peak strain.

**Figure 7:**
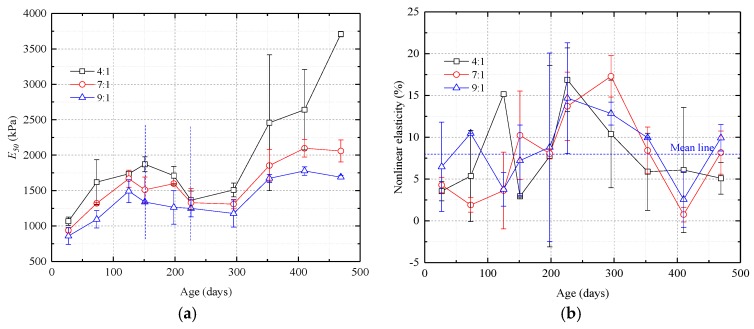
Secant modulus (*E*_50_) and elastic modulus gradient vs. time curves for samples with different proportions of silica sol and catalyst. (**a**) *E*_50_ curves. The curves can be divided into an ascending stage, a descending stage, and a second ascending stage; (**b**) Nonlinear elasticity curves. Throughout the course of the experiment, the nonlinear elasticity remains low. Before failure, the samples behave as linear elastic materials.

As shown in Figure 6 and Figure 7, the way in which UCS and *E*_50_ change over time are similar, although the magnitudes of the changes are different. The curves can be divided into three stages, an ascending stage, a descending stage, and a second ascending stage. In the first ascending stage, the first 150 days, UCS and *E*_50_ increase. In the descending stage, from around day 150 to days 200–230, UCS and *E*_50_ gradually drop. In the second ascending stage, UCS and *E*_50_ have increased since the 200–230-day time and the rate of increase is higher than that during the first ascending stage. The curves also show that more catalyst causes UCS and *E*_50_ to reach higher values.

The samples’ peak strain changed little with age and remained in the 4–8% range, only two percentage points above or below the average peak strain of 6%. This indicates that the samples could undergo significant deformation before they failed. The amount of catalyst also had little effect on peak strain.

Nonlinear elasticity of samples with different proportions of catalyst was between 0% and 18%, with an average of 8%. The results show that, before failure, the samples behave as linear elastic materials.

**Figure 8:**
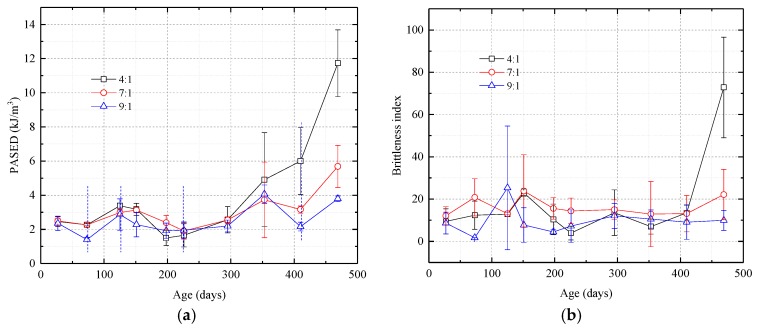
Graphs showing pre-peak absorption strain energy density (PASED) and brittleness index curves vs. time for samples with different silica sol:catalyst ratios. (**a**) PASED. The curves are W-shaped prior to 400 days. After 400 days, higher catalyst ratios result in significantly higher PASED; (**b**) Brittleness index. Samples with different silica sol:catalyst ratios have similar brittleness indexes before 400 days but after 400 days, samples with more catalyst have higher brittleness indexes.

As shown in Figure 8, before day 400, the PASED curves are W-shaped and are basically unaffected by the amount of catalyst. The two troughs are around day 75 and day 230 and the peak is around day 125. After day 400, however, PASED increases rapidly and the effect of the amount of catalyst on PASED becomes significant. Higher silica sol:catalyst ratios cause PASED to increase to higher values, and the rate of increase is more rapid.

As illustrated by Figure 8b, before 400 days of curing, the brittleness index of the samples was essentially unchanged with an average value of 12.40, a high brittleness. The sol:catalyst ratio had almost no influence on the brittleness index. However after 400 days of curing, the greater the amount of catalyst used in the grout mixture, the higher the brittleness index.

**Figure 9:**
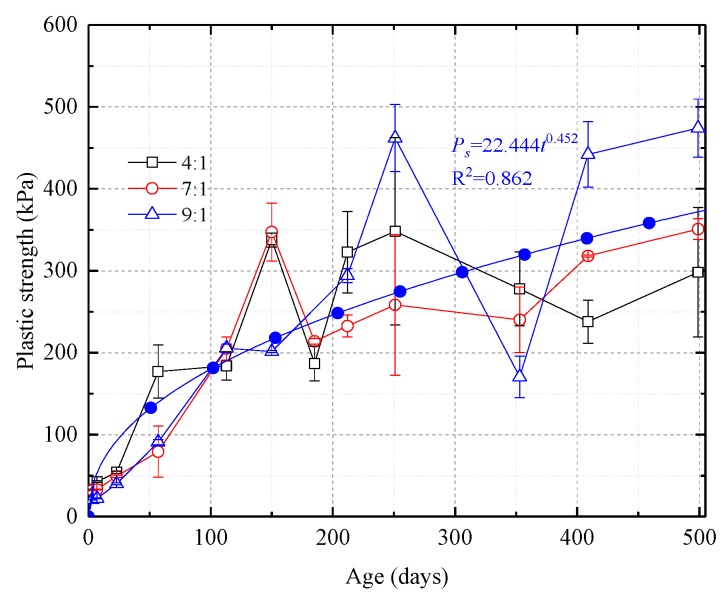
Plastic strength-time curves for samples with different proportions of silica sol and catalyst. The curves show allometric scaling for plastic strength.

As shown in Figure 9, the plastic strength-time curves for samples with different proportions of silica sol and catalyst exhibit allometric scaling. The equation for the line fit to the data in Figure 9 is shown below as Equation (2):

(2) $$P_{s}=22.444t^{0.452}$$

where, *P*_s_ is the plastic strength; *t* is the age.

Before 400 days, the effect of catalyst dosage on the grout’s plastic strength is not clear. After 400 days, the less the amount of catalyst used, the greater the grout concretion’s plastic strength.

## 4. Discussion

### 4.1. Weight- and Geometric Parameter-Time Law

The difference between this study and the study done by Axelsson (2006) is that in Axelsson’s experiments, the curing temperature was 8 °C, and the samples were cured under three controlled relative humidities, 75, 95, and 100%. Axelsson’s 180-day experiments showed that the greater the relative humidity, the less the drying shrinkage and that shrinkage tended to stabilize by day 180. In the experiment done for this paper, the relative humidity for the first 180 of the 500 days concentrated in the 70–90% range, and the temperature was usually between 20 and 30 °C. The drying shrinkage for our samples during this time is similar to that of Axelsson’s samples and the shrinkage also tends to stabilize at about day 180. In addition, as shown in Figure 4, less catalyst in the grout mix results in higher shrinkage. These results show that 180 days may be the curing time needed for initial drying shrinkage stability under high relative humidity (greater than 70%) and either normal (20–30 °C) or low (8 °C) temperatures. In Xuzhou City, where this study was conducted, the 200–300-day study interval was in the winter (November to February, Figure 2) and the average high temperature was about 8 °C. These temperatures essentially extended Axelsson’s (2006) experiment. In the 200–300-day period, the weight, height, diameter, and volume of the samples were fairly stable and this also indicates that 180 days might be the curing time for initial drying shrinkage stability. In this study’s second shrinkage stage, temperature was back up to the 20–30 °C range, reactivating moisture loss. Temperature rise is a driving force for moisture loss.

### 4.2. Strength- and Secant Modulus-Time Law and Micro-Mechanism

Butrón et al. (2009) showed that the strength of silica sol grout increased over time in 180-day experiments. As illustrated in Figure 6 and Figure 7, the values for UCS and secant modulus from samples in this study can be divided into an ascending stage, a descending stage, and a second ascending stage. The first ascending stage results are similar to Butrón et al.’s (2009) experimental results. The Butrón experiments also showed that samples that were cured for the same amount of time have greater strength when cured at higher temperatures under lower relative humidities. For the samples tested by this study, the weight and geometric parameters of the samples remained about the same in the descending stage, but in this study, the temperature decreased significantly and the relative humidity increased slightly. This suggests that the descending stage in this experiment was caused by the combined effects of temperature and humidity. In this study’s second ascending stage, temperature increased, relative humidity decreased slightly, and UCS and the secant modulus increased. The brittleness index also increased.

**Figure 10:**
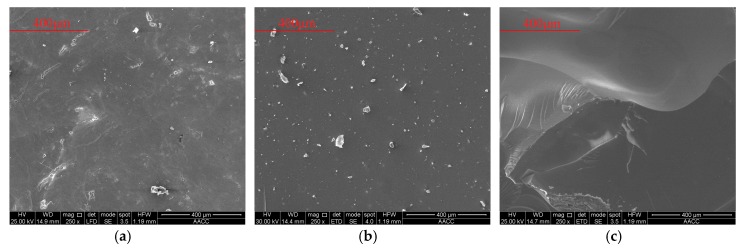
Scanning electron microscope (SEM) results for samples at three different times during the course of the experiment. (**a**) 2 d; (**b**) 206 d; (**c**) 500 d. The magnification of all is 250 times.

**Figure 11:**
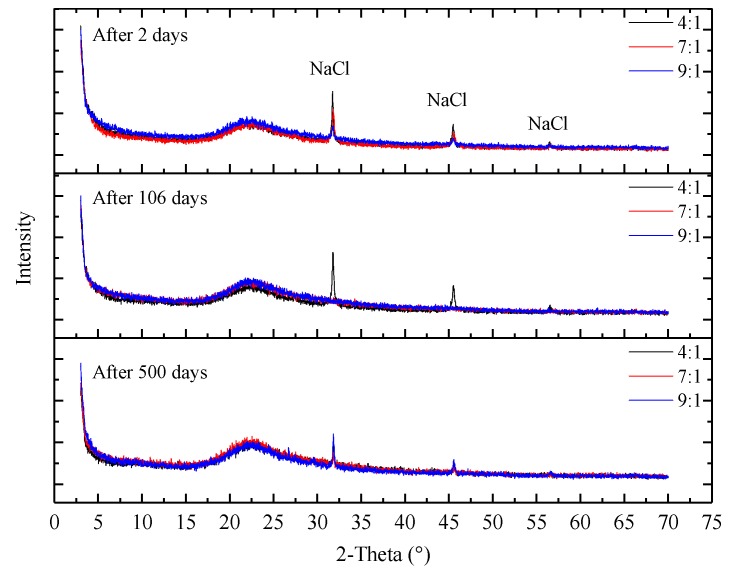
X-ray diffraction (XRD) results for samples with different proportions of silica sol and catalyst at three different times during the course of the experiment.

**Figure 12:**
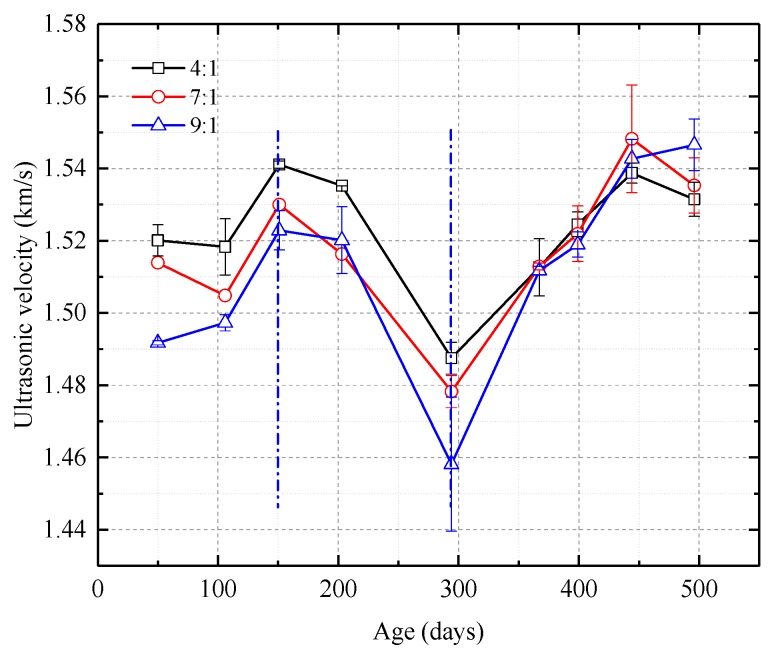
Ultrasonic velocity-time curves for samples with different proportions of silica sol and catalyst.

At the same time of mechanical tests, microstructure, crystal characteristics and compactionness experiments were carried out to analyze the strength change micro-mechanism of samples. As shown in Figure 10, the microstructure properties of samples were inspected with a FEI Quanta™ 250 scanning electron microscope (SEM) (Hillsboro, OR, USA). There are few pores in the initial stage, and there is no pore on the surface of the intermediate and later samples. Only a small number of irregular particles adhere to the surface of the sample. As shown in Figure 11, D8 Advance X-ray diffraction (XRD) (Bruker Corporation, Karlsruhe, Germany) showed that the crystal structure of samples are basically unchanged. As shown in Figure 12, C61 Ultrasonic Non-metal Detector (Sinotesting Technology, Beijing, China) showed that ultrasonic velocity-time curves for samples are similar to UCS curves. The ultrasonic velocity represents the compactionness. These indicate that the curing conditions mainly affect the compactness, and then affect the strength.

### 4.3. Plastic Strength-Time Law

When the Vicat cone is in static equilibrium, the calculated plastic strength includes shear strength from the grouting concretion on the sides of the cone and the grouting concretion’s compressive strength. Therefore, the plastic strength should be slightly larger than the unconfined compressive strength. This study also proves this relationship. The study also shows that as curing time increases, the plastic strength scales allometrically.

## 5. Conclusions

To study the long-term mechanical behavior of nano silica sol grout prepared with different proportions of catalyst in an environment of fluctuating temperature and humidity, a 500-day experiment has been carried out. Simultaneously, SEM, XRD, and ultrasonic velocity tests were carried out to analyze the strength change micro-mechanism of samples. The main conclusions from this experiment are:

(1) The temperature and humidity fluctuate over both long and short periods. As the dosage of catalyst in the grout mix is decreased, the curves showing the changes in sample weight and sample width, height, and volume over time can be divided into three stages, a shrinkage stage, a stable stage, and a second shrinkage stage. Higher amounts of catalyst improve the stability of the samples and reduce moisture loss. Temperature rise is also a driving force for moisture loss.
(2) The uniaxial compressive stress-strain curves all show that the samples are elastoplastic. The deformation can be divided into four phases, a compaction phase, an elastic phase, a plastic phase, and a post-peak stress phase. The curves for the uniaxial compression strength and the secant modulus can be divided into an ascending stage, a descending stage, and a second ascending stage. Peak strain for the samples changed little with curing time. The PASED-time curves are W-shaped and are essentially unaffected by the amount of catalyst prior to 400 days. After 400 days, higher catalyst ratios increase the PASED values significantly. Sample brittleness increases with time and in the later stages of the experiment, the brittleness index increases with higher catalyst dosages.
(3) Plastic strength-time curves for samples with different proportions of catalyst exhibit allometric scaling. A consistent effect of catalyst dosage on plastic strength is not apparent prior to 400 days of curing but after 400 days, it is clear that when the grout mix contains less catalyst, the plastic strength of the grout is greater.
(4) The surfaces of samples are smooth and compact at different ages, substantially unchanging. The crystal structure of samples are basically unchanged. Ultrasonic velocity-time curves for samples are similar to UCS curves. These indicate that the curing conditions mainly affect the compactness, and then affect the strength.

## Figures and Tables

**Table 1:** Basic parameters of the hydrolyzed silica sol and catalyst used in this study.

Properties	Silica Sol	Catalyst
Viscosity	~10 mPa·s	~1 mPa·s
Density	1.1 kg/L	1.07 kg/L
pH	10	7
Concentration (% by weight)	SiO_2_ 15%	NaCl 10%
